# Comparative Efficacy of Active Group Music Intervention versus Group Music Listening in Alzheimer’s Disease

**DOI:** 10.3390/ijerph18158067

**Published:** 2021-07-30

**Authors:** María Gómez-Gallego, Juan Cándido Gómez-Gallego, María Gallego-Mellado, Javier García-García

**Affiliations:** 1 Clinical Neuroscience Research Group, Faculty of Health Sciences, Catholic University of Saint Anthony, 30100 Murcia, Spain; 2 Applied Economics Department, Faculty of Economics, University of Murcia, 30100 Murcia, Spain; jcandido.gomez@um.es; 3 Espinardo Primary Health Care Centre, 30100 Murcia, Spain; maria.gallego3@um.es; 4 Clinical Neuroscience Research Group, Faculty of Economics, Catholic University of Murcia, 30107 Murcia, Spain; Jfggar@gmail.com

**Keywords:** Alzheimer’s disease, cognition, behaviour, daily living activities, motor function, active music intervention, receptive music intervention

## Abstract

Background: Music interventions are promising therapies for the management of symptoms in Alzheimer’s disease (AD). Globally, music interventions can be classified as active or receptive depending on the participation of the subjects. Active and receptive music tasks engage different brain areas that might result in distinctive clinical effects. This study aims to compare the clinical effects of two types of music interventions and a control activity. Methods: Ninety AD patients from six nursing homes participated in the study. Nursing homes were randomly and blindly assigned to receive either active music intervention, receptive music intervention, or the usual care. Effects on cognition, behaviour, daily living activities, and motor function were assessed. Results: Active music intervention improved cognition, behaviour, and functional state in a higher extent than both receptive music intervention and usual care. The effect size of active music intervention for cognitive deficits and behavioural symptoms was large (η^2^ = 0.62 and 0.61, respectively), while for functional state, it was small-to-medium sized (η^2^ = 0.18). Receptive music intervention had a stabilizing effect on behavioural symptoms compared to control intervention (mean change from baseline ± standard deviation = −0.76 ± 3.66 and 3.35 ± 3.29, respectively). In the active music intervention, the percentage of patients who showed improvement in cognitive deficits (85.7), behavioural symptoms (92.9), and functional state (46.4) was higher than in both receptive listening (11.8, 42.9, and 14.3, respectively) and control group (6.3, 12.2, and 17.1, respectively). Conclusions: Active music intervention is useful to improve symptoms of AD and should be prescribed as a complement to the usual treatment.

## 1. Introduction

Alzheimer’s disease (AD) is the most common neurodegenerative disease worldwide [1]. This disease is characterized by a progressive cognitive impairment and behavioural symptoms that cause loss of functional abilities and high socio-economic costs [2]. Nowadays, pharmacological approaches are aimed to reverse neurotransmitter deficits and ameliorate AD symptoms [3]. Rehabilitation strategies are focused on maintaining patients’ cognitive, motor, and functional skills. The effectiveness of these interventions requires patients to be adequately engaged and motivated to participate in the tasks and activities [4].

Music might be used for rehabilitation purposes since it may change the activity of many brain structures (e.g., related to sensory-motor processing, motivation, affect, attention, and memory) and induce plastic changes in some brain networks [5,6]. Additionally, at the neurochemical level, music stimuli influence the function of stress and arousal systems [7]. According to Thompson and Schlaug [8], music can be used to improve people’s health because of its ability to capture our attention, facilitate learning, modulate our emotions, stimulate body movement elicit memories, and promote social communication.

Patients with AD commonly find listening to music, singing, and dancing enjoyable and motivational [9], which increases subjects’ engagement in music-therapy sessions. Furthermore, there is some evidence about the benefits of music on anxiety and agitation in patients with AD [10,11,12]. In fact, relaxing music induces an increase in serum melatonin levels that may contribute to participants’ calm mood [12]. However, other studies reported that these effects might not be stronger than those obtained with other recreational activities [13].

As cognitive functions are concerned, improvement in self-consciousness, orientation, language, autobiographical memory, and global cognition have been reported following music-based interventions [14,15,16]. There is controversy about the duration and the magnitude of such effects [17,18]. This can be partly explained by heterogeneity among studies regarding the professional who led the intervention, the type of music-based intervention, and even the clinical context [18].

Music-based interventions range from music therapy, “a clinical and evidence-based use of music interventions to accomplish individualized goals within a therapeutic relationship by a credentialed professional” [19], to other kinds of music-based practices developed by people with different degrees of music training. Music therapy has been suggested to be more effective in the alleviation of mood disorders than other types of music interventions [20].

Concerning the degree in which recipients are involved in creating music, music interventions can be classified as active and receptive. In active music-based interventions, a facilitator encourages participants to express their emotions by creating musical sounds and rhythms. These interventions usually enable the development of a stronger interpersonal relationship between the person and the facilitator [21]. On the contrary, receptive techniques, usually based on music listening, involve the client responding verbally or in another modality. The aim of receptive techniques is generally to evoke an emotional response or memories and stimulate self-knowledge. Besides, as these interventions require a lower level of participation, they are potentially useful even in late stages of the disease. In fact, the ability to recognize and remember music is relatively preserved in AD patients [22].

Currently, there is still a paucity of studies comparing active versus receptive music-based interventions. In patients with mild-to moderate dementia, Sarkämo et al. [23,24] found that although both types of music intervention (singing and music listening) are effective for cognitive and depressive symptoms, the pattern of improvement may be different between them. Moreover, these effects may be influenced by demographic and clinical characteristics of patients with dementia [25]. In moderate-severe dementia, Sakamoto et al. [10] reported that both kinds of intervention (active and receptive) have relaxing effects by parasympathetic activation, but active music intervention caused a greater reduction in behavioural disorders. In this sense, Raglio et al. [26,27] reported higher effects of active music therapy than music listening on behavioural symptoms although the results did not reach statistical significance. However, a recent meta-analysis supports that receptive music interventions may be more effective than active methods in reducing anxiety, agitation, and other behavioural symptoms [28].

This study aims to compare the effects of two types of group and preferred music-based interventions (active and receptive) with a control activity on cognition, behaviour, motor function, and abilities. Unlike other studies in people with several types of dementia, the present study was performed in patients with AD in a mild or moderate stage. We hypothesized that both music-based interventions would have beneficial effects compared to control intervention and that active music intervention would be more effective than receptive music intervention. 

## 2. Materials and Methods

### 2.1. Participants

The participants were recruited from six nursing homes in the Region of Murcia. A nursing home was selected if it was expected to have at least ten eligible residents. Inclusion criteria were diagnosis of probable AD and mild or moderate stage of the dementia (Clinical Dementia Rating) [29]. Exclusion criteria included aphasia and hearing impairment that might affect participation in the activities. All residents that met the eligibility criteria and gave informed consent to participate were finally selected. All of them were receiving pharmacological therapy and cognitive stimulation therapy in the nursing homes. All clinical data (diagnosis, level of severity, and treatment) were obtained from medical records. The study protocol was approved by the Ethical Committee of Catholic University of Saint Anthony (code 111656).

### 2.2. Research Design, Procedure, and Interventions

The study has been registered (ID: NCT04761497). The effects of three types of intervention were compared. The design is quasi-experimental. Each nursing home, defined as a cluster, was randomly assigned to receive either active music intervention (AMI), receptive music intervention (RMI), or control intervention. Clusters (nursing homes) were allocated 1:1:1 among trial arms. Such randomization was performed by an independent external researcher. Statistical analysis was performed by a professional blinded to residents’ data. 

To know residents’ musical preferences, the music facilitators (M.G.-G. and M.G.-M.), both with master’s-level qualification in creative arts therapy and specialization in music therapy and co-authors of this study, administered the questionnaire of musical preferences to them [30]. This instrument includes questions about the preferences for musical genres, singers, instruments, and songs. In our sample, 95.9% of the residents liked boleros, 79.6% liked sevillanas, and 57.1% liked religious music. On the contrary, 44.9% of residents did not like classical music, and 36.7% did not like opera. Considering such preferences, the music facilitators developed 3 lists of 12 songs each. The duration of each song was limited to 3 min. The same lists of songs were used for both music interventions with the exceptions of the welcome and goodbye songs, which were replaced by two different songs in receptive music groups. Each intervention lasted approximately 45 min and was performed twice a week for three months (12 sessions in total). The list of songs was changed each month. Rooms where music activities took place were soundproofed and spacious enough for the residents to be comfortable. These settings and those corresponding to the control activities were kept constant for all sessions. The facilitator was also kept constant for the same group.

Active Music Intervention (AMI)

There were four groups of 6, 7, 8, and 9 residents that received AMI sessions. A typical session consisted of:

1. Welcome song. At the beginning of each session, residents had to greet and introduce themselves. This activity was selected to create an atmosphere of trust and facilitate residents’ interaction.
2. Rhythmic exercises. For this activity, three well-known songs were used. The music facilitator and the residents had to keep the songs’ rhythm by clapping their hands each time they heard the chorus. For example, in the song “Quizás, quizás, quizás”, by Nat King Cole, residents had to clap their hands each time the singer says “quizás.”
3. Dance exercises. In this activity, three songs were used. The facilitator encouraged residents to make free body movements in response to music and facilitated residents dancing by reflecting or amplifying their spontaneous movements. Besides, they tried to engage residents more reluctant to participate by talking to them or making eye contact.
4. Music quiz. For this task, the residents were divided into two teams. A list of four songs was played. Each team should guess the songs’ names. Then, the facilitator encouraged residents to name the singer as well. Finally, the facilitator and the residents sang the chorus of the song. This activity aimed to facilitate interaction among residents as well as improve semantic and autobiographical memory.
5. Goodbye song. At the end of each session, residents said goodbye to the group.

The objectives of the activities being programmed were to improve cognition (activities 2 and 4), attention, synchronization, and movement fluency (activities 2 and 3), socialization (activities 1, 3, 4, and 5), and mood (activities 3 and 4). When necessary, facilitators adapted their interventions to the needs of residents to avoid adverse reactions.

Receptive Music Intervention (RMI).

There were three groups of residents receiving RMI, with 6, 7, and 8 people each. Residents of these groups and the facilitators were comfortably seated in a medium-sized room while listening to the playlist previously recorded in a computer. At the beginning and at the end of each session, the facilitator briefly assessed the needs of the group and selected a song accordingly. After each song, the facilitator told the residents the song title and the singer’s name and allowed residents to share their feelings or memories. Besides, some residents spontaneously clapped, tapped feet, or nodded along with music, but they did not have direct participation in the music. 

The aims of this intervention were to improve mood, anxiety, and socialization.

Control Activity

There were four groups of residents receiving control activity, with 8, 9, 11, and 12 residents each. In a large-sized room, residents were seated watching nature videos. These videos were documentary films about African animals, with the nature sounds but without music. Such videos have nearly the same duration as the music interventions. The facilitator and two nurses were with them facilitating the activity.

After each music intervention, the facilitator registered the participation of residents. In any activity, when residents showed disturbing behavioural symptoms, the facilitator tried to respond to their needs to minimize the influence on the group atmosphere. Residents with disruptive behaviours that could not be reversed were temporarily excluded from the sessions. 

### 2.3. Outcome Measures

Mini Examination of the Mental State (MMSE) [31]. This instrument was selected to assess changes in global cognition because it is one of the most widely used brief cognitive tests for dementia and is easy to administer. Total scores range from 0 to 30, with higher scores indicating better global cognition. A version validated in the Spanish population by Blesa et al. [32] was used. As described in the former study, MMSE scores were corrected by age and educational level. 

Neuropsychiatric Inventory (NPI) [33]. A 12-item nursing home version was used to assess changes in behavioural disorders. This is an interview-based instrument that assesses the frequency and severity of 12 neuropsychiatric symptoms: delusions, hallucinations, agitation/aggression, dysphoria/depression, anxiety, euphoria/elation, apathy, disinhibition, irritability, aberrant motor behaviour, night-time behavioural disturbances, and appetite/eating disturbances. The frequency of each symptom is rated from 1 to 3 (being 3 the commonest) and the severity from 1 to 3 (being 3 the severest). The score of each symptom is obtained by multiplying frequency and severity. The total NPI score is the sum score of all symptoms’ scores (range: 0–144). 

Geriatric Depression Scale (GDS) [34]. A 15-item version of this instrument was used to assess the effect of the interventions on residents’ affective state. This version has been validated in patients with dementia [35]. Possible scores range from 0 to 15, with higher scores indicating more depressive symptoms. 

Barthel Index (BI) [36]. This 10-item instrument was used to evaluate changes in the ability to do basic daily living activities. The BI total score is obtained by summing each item score, and it ranges from 0 (highest dependence) to 100 (independence). The validity of the Spanish version has been reported [37].

Tinneti Scale (TS) [38]. This scale was used to assess the effects of the interventions on residents’ motor function. It consists of two subscales: balance test (9 items) and gait test (8 items). Items are scored from 0 to 1 or 2, with higher scores indicating better function. Total score of TS is the sum of the balance test’s score (range 0–16) and gait test’s score (range 0–12).

Before the intervention and just after it, trained nurses administered residents the MMSE, GDS, and TS and completed NPI and BI.

### 2.4. Statistical Analysis

Differences among groups in demographics and baseline scores in the outcome measures were assessed by analysis of variance (ANOVA). Paired *t*-test was used to assess the change from the baseline in outcome measures scores. To examine differences in efficacy, the adjusted mean change from the baseline in each outcome measure was compared among groups by analysis of covariance (ANCOVA), with baseline scores as covariates. In addition, differences in the percentage of responders between groups, defined as an improvement in the outcome measures scores, were assessed with χ^2^ test. All the statistical analyses were performed using the software SPSS version 19.

## 3. Results

### 3.1. Baseline Assessment

The sample consisted of 90 AD residents. Of these subjects, 28 were in the AMI group (71.5% women), 21 were in the RMI group (61.9% women), and 41 were in the control group (54.5% women). The mean age of the AMI group was higher than that of the RMI and control group (Table 1, *p* < 0.001 for both contrasts). As civil status is concerned, 47.72% of the residents were married, 34.44% were widowers, and 17.78% were single. According to the CDR scale, 70% of the residents were mild and 30% were moderate.

According to Hair [39], the distribution of the data can be accepted as normal if skewness 95% confidence interval (SCI) is between −2 to +2 and kurtosis 95% confidence interval (KCI) is between −7 and 7. Variables normally distributed were: age (SCI = −0.60–0.40; KCI = −1.70–0.83), years of education (SCI = −0.60–0.40; KCI = −1.12–0.88), MMSE (SCI = −0.37–0.64; KCI = −1.59–0.42), BI (SCI = −0.10–0; KCI = −1.00–1.00), TS (SCI = −0.05–0.10; KCI = −0.40–1.00), NPI (SCI = −0.10−1.10; KCI = −0.19−0.38), and GDS (SCI = −0.13–0.87; KCI = −1.76–0.24). Groups were comparable in educational level, cognition, functional ability, motor function, depression, and neuropsychiatric symptoms (*p* > 0.05). The average number of sessions per resident ranged from 18–19 in the AMI group, 19–20 in the RMI group, and 19–21 in the control group. Only one resident (from the AMI group) discontinued the intervention because she transferred to another facility.

### 3.2. Efficacy of Music-Based Interventions

**Figure 1:**
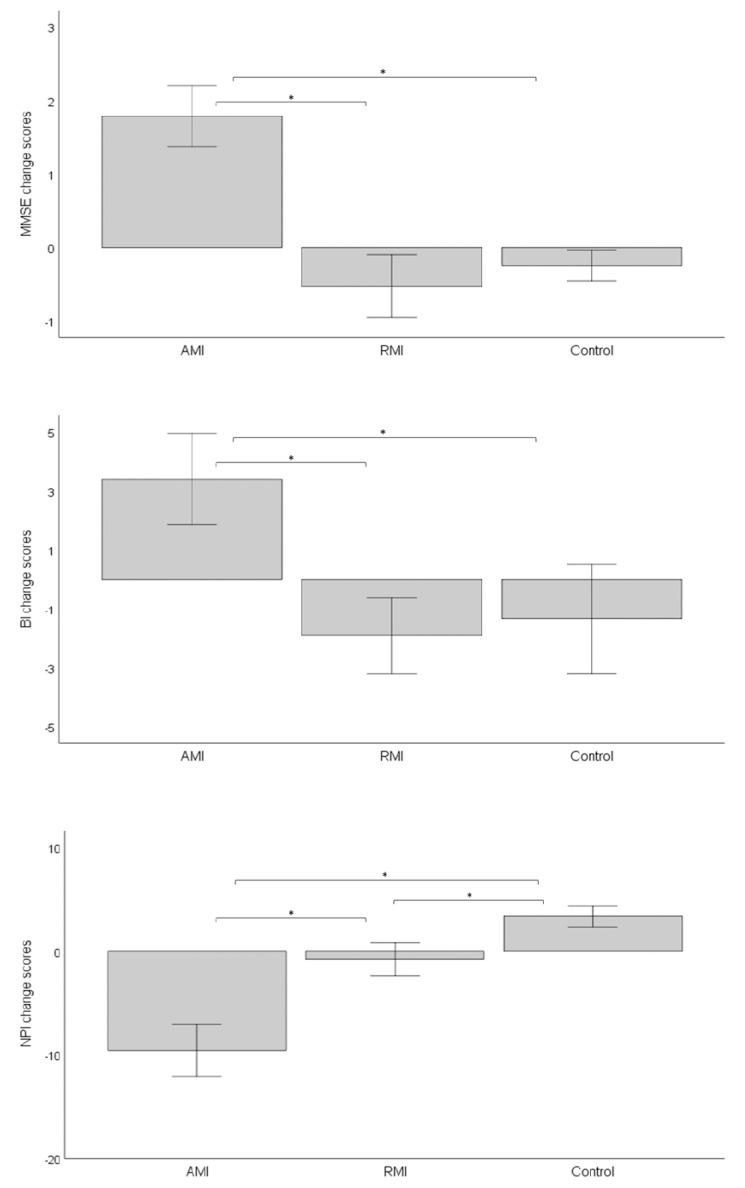
Graphical representation of the change in MMSE, NPI, and BI scores by group. AMI, active music intervention; BI, Barthel index; MMSE, Mini Examination of the Mental State; NPI, Neuropsychiatric Inventory; RMI, receptive music intervention. Bars represent standard error * *p* < 0.001 in post hoc comparison after one-way ANCOVA.

**Figure 2:**
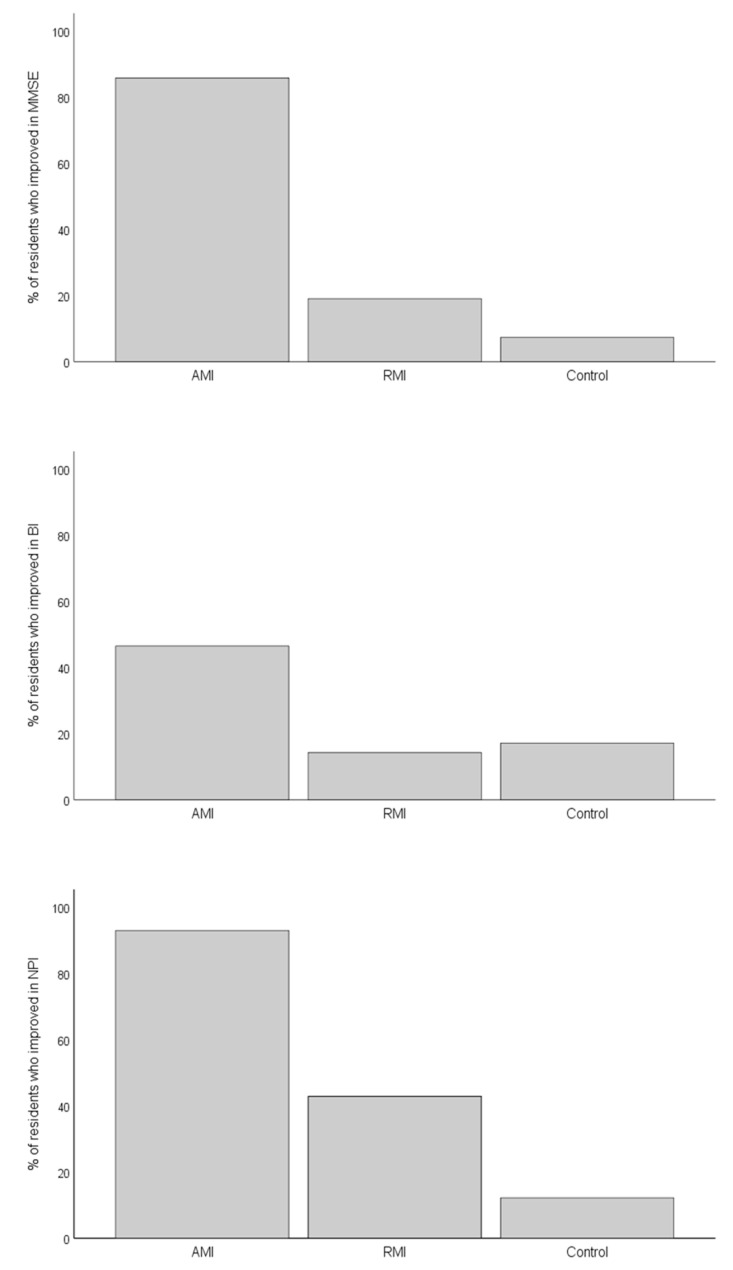
Graphical representation of the percentage of residents who improved in MMSE, NPI, and BI by group. AMI, active music intervention; BI, Barthel index; MMSE, Mini Examination of the Mental State; NPI, Neuropsychiatric Inventory; RMI, receptive music intervention. *p* < 0.001 in χ^2^ test.

After the intervention, mean MMSE scores significantly increased in the AMI group while decreasing in the RMI and in the control group (Table 2). Accordingly, ANCOVA showed significant differences in MMSE change scores among intervention groups (F (2,89) = 68.7; *p* < 0.001; η^2^ = 0.62). No significant association was found between basal MMSE scores and MMSE change scores (*p* > 0.1). As shown in Figure 1, mean MMSE change scores were significantly higher in AMI group than in both the RMI and the control groups (Bonferroni corrected *p* < 0.001 in both cases). In addition, the percentage of residents who improved in MMSE scores was higher in the AMI group than in either other group (Figure 2, *p* < 0.001 for both contrasts), while no difference was found between the RMI and control groups (*p* = 0.214). 

Concerning functional abilities, mean BI scores significantly increased in the AMI group, decreased in the RMI group, and did not change in the control group (Table 2). ANCOVA confirmed differences in mean BI change scores among groups (F (2,89) = 9.66, *p* < 0.001, η^2^ = 0.18). Basal BI score was not associated with BI change scores (*p* < 0.100). Post hoc analyses showed that mean BI change scores were significantly higher in the AMI group than in the other groups (*p* < 0.001 in both cases, Figure 1). Besides, the percentage of residents that improved in BI scores was significantly higher in AMI group than in both the RMI group (*p* = 0.003) and the control group (*p* = 0.014) (Figure 2). 

As shown in Table 2, after the intervention, a significant increase in mean TS score was observed in the AMI group as opposed to the RMI and control groups. However, ANCOVA revealed that there was not a significant difference in mean TS change scores among groups (F (2,89) = 2.27; *p* = 0.110). 

As to behavioural disorders, mean NPI scores significantly decreased in the AMI group, did not change in the RMI group, and increased in the control group after the intervention (Table 2). ANCOVA confirmed significant differences in mean NPI change scores among groups (F (2,89) = 67.3; *p* < 0.001; η^2^ = 0.61). No relationship between basal and change NPI scores was found (*p* > 0.100). Post hoc tests revealed that mean NPI change scores were lower in the AMI group than in the other groups (*p* < 0.001 for both contrasts) and lower in the RMI group than in the control group (*p* = 0.004) (Figure 1). As Figure 2 shows, the percentage of residents that showed a reduction in NPI symptoms was significantly higher in the AMI group than in either other group (*p* < 0.001 for both contrasts) and was higher in the RMI group than in the control group (*p* = 0.010).

No significant change in GDS mean scores was observed after the intervention in any of the groups (Table 2). 

## 4. Discussion

The results of this study support the beneficial effects of the music-based interventions for AD symptoms. Particularly, AMI was found to improve symptoms of three main clinical domains of AD (cognition, behaviour, and functional state) compared to control intervention, while RMI had a stabilizing effect on neuropsychiatric symptoms. Interestingly, both cognitive and neuropsychiatric effects of AMI were large even after controlling for baseline scores. 

Our findings about the positive effects of AMI on behaviour agree with those previously reported in patients with severe AD [11] and in patients with dementia [23,25,26,27]. However, in RMI group, neuropsychiatric symptoms remained stable. This result was unexpected, as listening to music, especially if based on the person’s preferences, might trigger positive memories that contribute to relaxation [40]. This could be because behavioural symptoms of RMI group were not as severe as in the other groups although the contrast did not reach statistical significance. 

No improvement in residents’ affective state was observed with any music intervention in contrast to others reported with AMI [25] and music listening [10,40]. Affective state remained stable in the three groups. Perhaps this finding might be due to personal attention and care provided by staff and the residents’ knowledge of participating in a study. Additionally, the lack of an intervention effect could be attributed to either the inclusion of residents with low GDS scores or to the wide variability of the data. We did not examine the differences in the use of anxiolytic or neuroleptic drugs before and after the intervention; thus, we cannot rule out that the use of psychotropic drugs might have changed during this study.

The effects on cognition of active music interventions in residents with AD are in concordance with those previously reported [14,15,16]. The use of preferred music genres might have contributed to the large size effect observed since it might have improved autobiographical memory [41,42]. However, no effect on cognitive functions was observed in the music-listening group. One possible explanation for this finding might be that music creation itself acts as a memory enhancer [43]. Since active music tasks, like singing or playing instruments, are cognitively more demanding in terms of memory, verbal processing, or motor planning, they are more likely to improve these functions than music listening [44]. In addition, AMI is likely to promote socialization, engagement, and self-expression in a higher degree than other interventions, involving greater recruitment of task-related and additional brain regions [45]. Moreover, in AMI sessions, where subjects are asked to make body movements along to the music, an enhancement of music perception may be observed as reported [46]. Dancing may be understood as an example of a combined intervention involving physical and cognitive resources and induced brain plasticity [47]. In fact, dance-movement therapy has been suggested to improve cognition and behaviour of patients with AD [48]. Thus, the small improvements observed in balance, gait, and functional abilities might be explained by the inclusion of the dance activity in the AMI. 

This study has several strengths, namely: (i) inclusion of an active control group consisting of residents participating in a relaxing activity; (ii) comparison between two types of music intervention; and (iii) using several outcome measures. Nevertheless, it has some limitations that should be considered. Changes in psychotropic treatment were not controlled. Information bias is possible since the administration of the instruments was not blinded. Besides, there might be a selection bias, as the participants were not selected from the same sample. Due to its quasi-experimental design, the equivalency among the groups cannot be guaranteed for any variable, which limits the generalizability of the study. The associations shown should be tested in future studies. 

## 5. Conclusions

Current findings show that adding AMI to the usual treatment may improve cognition, behaviour, and dependence of mild-to-moderate AD residents. Instead, RMI has only a stabilizing effect on behaviour. Thus, whenever possible, AMI should be preferred over RMI in people with AD.

## Figures and Tables

**Table 1:** Baseline characteristics of AD residents in the groups of intervention and in the control group.

	AMI (*n* = 28)		RMI (*n* = 21)		Control (*n* = 41)		*p*-Value
	Mean	SD	Mean	SD	Mean	SD	
Age	83.93	8.01	78.67	5.73	80.02	5.78	0.009
ED	7.28	1.04	7.04	1.74	7.48	1.93	0.081
MMSE	17.79	3.90	18.28	6.14	19.95	3.19	0.094
NPI	20.92	9.20	18.38	7.48	24.53	10.91	0.053
BI	70.89	13.88	70.71	17.34	77.93	12.39	0.065
TS	12.25	2.74	11.67	1.71	11.39	1.61	0.235
GDS	6.71	1.60	5.47	1.93	5.88	1.87	0.051

AMI, active music intervention; BI, Barthel index; ED, education (years); GDS, Geriatric Depression scale; MMSE, Mini Examination of the Mental State; NPI, Neuropsychiatric Inventory; RMI, receptive music intervention; SD, standard deviation; TS, Tinetti Scale.

**Table 2:** Scores of the outcome measures before and after the interventions.

		Basal		Final			
	Group	Mean	SD	Mean	SD	t	*p*-Value
MMSE	AMI	17.79	3.90	19.57	3.80	8.548	<0.001
	RMI	18.28	6.14	17.57	6.14	3.873	<0.001
	Control	19.95	3.19	19.83	3.31	1.954	0.058
NPI	AMI	20.92	9.20	11.36	4.01	7.665	<0.001
	RMI	18.38	7.48	17.62	8.27	0.954	0.351
	Control	24.53	10.91	27.90	13.14	6.533	<0.001
BI	AMI	70.89	13.88	74.29	12.96	−4.385	<0.001
	RMI	70.71	17.34	68.81	18.43	2.961	0.008
	Control	77.93	12.39	76.59	13.76	1.451	0.155
TS	AMI	12.25	2.74	13.39	2.64	−8.034	<0.001
	RMI	11.67	1.71	11.57	1.75	0.525	0.605
	Control	11.39	1.61	11.63	3.48	0.522	0.604
GDS	AMI	6.71	1.60	6.46	1.55	1.491	0.148
	RMI	5.47	1.93	4.95	1.28	1.714	0.102
	Control	5.88	1.87	5.85	1.84	0.255	0.800

AMI, active music interventions; BI, Barthel Index; GDS, Geriatric Depression Scale; RMI, receptive music intervention; MMSE, Mini Examination of the Mental State; NPI, Neuropsychiatric Inventory SD, standard deviation; TS, Tinetti scale.

## Data Availability

Data is contained within the article.
